# Diverse subterranean fungi of an underground iron ore mine

**DOI:** 10.1371/journal.pone.0234208

**Published:** 2020-06-04

**Authors:** Benjamin W. Held, Christine E. Salomon, Robert A. Blanchette

**Affiliations:** 1 Department of Plant Pathology, University of Minnesota, St. Paul, Minnesota, United States of America; 2 Center for Drug Design, University of Minnesota, Minneapolis, Minnesota, United States of America; Friedrich Schiller University, GERMANY

## Abstract

Mines and caves are unusual ecosystems containing unique fungi and are greatly understudied compared to other environments. The Soudan Mine in Tower, MN, an iron ore mine that closed in 1963 after operating for 80 years, was sampled to explore fungal diversity and to investigate taxa that tolerate heavy metals for potential bioprocessing technologies or as sources of bioactive molecules for drug discovery and possible biocontrol for white-nose syndrome (WNS) of bats. The mine is 714 m deep, has 18 levels and contains large quantities of wooden timbers, in contrast to many other oligotrophic subterranean environments. Fungi were cultured from samples and the ITS region was sequenced for identification and phylogenetic analysis. Results show Ascomycota are the dominant fungi followed by Basidiomycota and Mucoromycota. Out of 164 identified taxa, 108 belong to the Ascomycota and 26 and 31 to Basidiomycota and Mucoromycota, respectively. There are also 46 taxa that do not match (<97% BLAST GenBank identity) sequenced fungal species. Examples of the most commonly isolated Ascomycota include *Scytalidium* sp., *Mariannaea comptospora*, *Hypocrea pachybasidioides*, *Oidiodendron griseum* and *Pochonia bulbillosa*; Basidiomycota include *Postia* sp., *Sistotrema brinkmannii*, *Calocera* sp., *Amylocorticiellum* sp.; Mucoromycota include *Mortierella parvispora*, *M*. *gamsii*, *M*. *hyaline*, *M*. *basiparvispora* and *Mortierella* sp. Unusual growth forms were also found including large quantities of black rhizomorphs of *Armillaria sinapina* and white mycelial cords of *Postia* sp. mycelium, as well as *Pseudogymnoascus* species growing over large areas of mine walls and ceiling. The mine environment is a relatively extreme environment for fungi, with the presence of high levels of heavy metals, complete darkness and poor nutrient availability. Several genera are similar to those isolated in other extreme environments but phylogenetic analyses show differences in species between these environments. Results indicate this subterranean environment hosts a wide diversity of fungi, many of them not found in above ground environments.

## Introduction

Fungi that inhabit subterranean environments are poorly understood and little is known about the microorganisms that dominate these unusual environments in comparison to other ecosystems. Cave and mine environments can be very different from other terrestrial sites, which have total darkness, relatively low temperatures, scarce or patchy organic matter, and often elevated concentrations of heavy metals [1]. These conditions can be limiting factors for microbial growth or act as selection pressure to allow fungi capable of functioning in an extreme environment to flourish. An early published reference of subterranean fungi was made in 1674 from a report of microbial growth in a coal mine in England [2], where large quantities of the “fungus subterraneus” was collected. The communication lacks a detailed description but it likely refers to Basidiomycota mycelium. Detailed descriptions of subterranean fungi were also presented in 1793 by Alexander Humboldt. He found unusual underground “plants” (now known to be fungi) in the mines of Freiburg, Germany [3]. Included in his report are drawings of fungi that suggest possible descriptions of *Trametes*, *Boletus* and *Calocera*. Humbolt also described a fungus called *Lichen verticillatus* or *Rhizopmorpha verticillatus*, from which drawings and description indicate it may be an *Armillaria* species. Since then, many other Basidiomycota have been identified from mines including *Fibroporia vaillantii*, *Serpula lacrymans*, *Coniophora puteana*, *Heterobasidion annosum*, and *Armillaria mellea* [4–7]. Recently, a review on the diversity of fungi in caves and mines with information from numerous studies revealed a diverse assemblage of fungi, slime molds and yeasts (1029 species), and that the majority of the taxa belonged to the Ascomycota (69.1%) followed by the Basidiomycota (20%) and the Zygomycota (6.6%) [8]. With the North American introduction of the fungal bat pathogen *Pseudogymnoascus destructans* (*Pd*) causing white-nose syndrome (WNS) that has decimated bat populations, many more studies have focused on microbial diversity of subterranean environments (caves/mines) in search of WNS antagonists for possible control measures and to elucidate *Pd* ecology [9–12].

The Soudan iron mine in Soudan, Minnesota was the first iron mine in the state and operated from 1884 until 1962. After its closure, it was gifted to the state of Minnesota and converted into the Soudan Underground Mine State Park in 1963 and operates mine tours for visitors that take them to the bottom level of the mine. The mine has 18 accessible levels with drifts (tunnels) in east and west directions from the main shaft. The mine is 713 m deep with one main shaft in operation and other closed shafts that were used during mining operation. Most subterranean environments such as natural karst caves and mines are oligotrophic [1,13,14]. However, in the Soudan mine an abundance of wood remains from mining activities, including thousands of timbers that supported rails for ore carts, beams and lumber for various purposes. This substrate provides a carbon and nutrient source for fungi and other microbes not usually found in subterranean cave environments. The Soudan Mine environment is also known to have elevated levels of heavy metals [15]. The mine represents an extreme environment in which microbial diversity has not been previously studied. The main objective of this study was to describe the fungal diversity associated with wood and other substrates found in the mine. In addition, while these studies were in progress, WNS was confirmed in bat populations in the mine and fungi isolated were screened for potential antagonistic effects against WNS fungi for bio-control in other studies. Fungi that can grow in this extreme environment of high metal ion concentrations were also of interest for bioprocessing technologies such as heavy metal remediation from mine wastewater and for bioactive compounds.

## Materials and methods

Samples were collected from 14 accessible levels of the Soudan iron mine in Lake Vermilion Soudan Underground Mine State Park in Tower Minnesota (in 2013, ‘14, ‘15, ‘17) and taken from wooden timbers and other wood materials as well as from visible fungal mycelia or rhizomorphs. Samples were placed in sterile whirl-pak bags and kept cold while being transported from the mine to the laboratory. Pure fungal cultures were obtained by aseptically placing four subsample segments of wood on two types of media, (MEA amended with 0.1 g/L streptomycin sulphate added after autoclaving and a semi-selective media for Basidiomycota which consisted of 15 g malt extract, 15 g agar, 2 g yeast extract, 0.06 g/L of Benomyl and 0.1 g/L streptomycin sulphate added after autoclaving) in duplicate. Plates were incubated at 22°C and once growth appeared, pure cultures were transferred to MEA plates. A culture of each morphotype isolated from each sample was then used for DNA extraction and sequencing. Pure cultures are stored in the University of Minnesota Forest Pathology culture collection. Sample collection was carried out under permit from the State of Minnesota Department of Natural Resources, Division of Parks and Trails in Lake Vermilion Soudan Underground Mine State Park.

Fungal DNA was extracted using a CTAB extraction procedure as previously described [16]. The internal transcribed spacer gene region (ITS) was amplified using primers ITS1F and ITS4 [17]. PCR was carried out in 25 μl reactions which contained ~12ng of DNA template, .25 μM forward primer, 0.25 μM reverse primer, 0.05 μg/μL BSA, 1X GoTaq® green mastermix and nuclease free sterile water. Thermocycler program parameters for amplification were: 94°C for 5 min, then 35 cycles of 94°C for 1 min, 50°C for 1 min, and 72°C for 1 min and a final extension at 72°C for 5 min. Amplicons were verified by electrophoresis on a 1% agarose gel with SYBR green 1 pre-stain and imaged with a Dark Reader DR45 (Clare Chemical Research–Denver, CO). Sanger sequencing was done with PCR primers on an ABI 3730xl DNA sequencer (Applied Biosystems–Foster City, CA). Consensus sequences were assembled using Geneious 9.0 [18] and were used to with the BLASTn program [19] using the megablast option in GenBank. Identification of cultures was based on the highest BLAST match score of a genus-species accession from a taxonomic study. Sequences representative of each taxon were deposited in GenBank and given generic or higher classification. Values of less than 97% best BLAST match to a verified genus / species were considered a possible new species [20,21]

Phylogenetic relationships of the cultures obtained were also determined using Geneious 9.0, while MAFFT v7.222 and MrBayes 3.2.6 plugins were used for sequence alignment and Bayesian analysis, respectively. jModelTest 2.1.10 [22] was used to determine the appropriate model (JC69) for Bayesian analysis. 1.1 x 10^6^ MCMC generations were used with a sampling frequency every 200 generations and the first 10% of sampled trees were discarded as burn in.

Elemental analyses were carried out on wood and *Armillaria* rhizomorphs using inductively coupled argon plasma optical emission spectrometry (ICP). Subsamples were taken from small wood samples from different locations in the mine for the elemental analyses. Sound pine and oak, cut from modern boards, were used as controls. Al, B, Ba, Be, Ca, Cd, Co, Cr, Cu, Fe, K, Li, Mg, Mn, Mo, Na, Ni, P, Pb, Rb, Si, Sr, Ti, V and Zn are determined simultaneously by Inductively Coupled Plasma Atomic Emission Spectrometry (ICP-AES). A 500 mg sample of dried plant material was weighed into a 20 mL high form silica crucible and dry ashed at 485°C for 10–12 hours. (Crucibles are covered during the ashing as a precaution against contamination.) The ash was equilibrated with 5 mL of 20% HCl at room temperature for ½ hour. Then 5 mL of deionized water was added, gently swirled and allowed to settle for 3 hours. The solution was decanted into 15 ml plastic disposable tubes for direct determination by ICP-AES [23–25].

## Results

The Soudan mine environment is dark, generally 4–12°C, and most levels have water seepage creating wet conditions. Thousands of wooden timbers have been left in the mine from past use for rail tracks, supports and other mining activities. These substrates provide a carbon source and nutrients for fungi to grow. Mine personnel indicated that the timbers used in the mine came from surrounding forests and the wood identified was mainly pine (*Pinus*) and spruce (*Picea*). Most of the woods sampled were soft, spongy and had advanced stages of decay.

The identification to species is based on the best BLAST match and for taxa matching less than 97%, the species listed may not be correct until further characterization is completed. A complete list of fungi is presented in S1 Table. From a total of 370 samples, 164 fungal taxa were isolated and identified by sequencing. This included 108 taxa in the Ascomycota, 26 in the Basidiomycota and 31 in the Mucoromycota (S1 Table). Forty-six, or 28% of the isolated taxa, have a best BLAST match to a described species below 97% identity, which may represent new species. Among these low percent matched taxa, 26 are in the Ascomycota, 9 are in the Basidiomycota and 10 in the Mucoromycota. Some of the most frequently isolated fungi are shown in Table 1 (only fungi isolated more than 10 times are listed with the number of isolations in parenthesis). Those in the Ascomycota include *Scytalidium* sp. (38), *Mariannaea camptospora* (22), *Hypocrea pachybasidioides* (20), *Oidiodendron griseum* (15), *Pochonia bulbillosa* (13), *Penicillium spinulosum* (12) and *Cosmospora viridescens* (10). The most encountered in the Basidiomycota were *Postia* sp. (26), *Sistotrema brinkmannii* (26), *Calocera* sp. (24), *Amyloathelia* sp. (11) and the most common Mucoromycota were *Mortierella parvispora* (27), *M*. *gamsii* (14) and *M*. *hyalina* (12).

**Table 1:** List of fungal taxa, the best BLAST match and frequency isolated at specific levels of the Soudan mine. Fungi isolated 5 times or more are listed. S1 Table lists all fungi isolated and GenBank accession numbers. Taxa appearing more than once indicate different strains with different % BLAST matches.

Taxa	% Match	Level														Total
		7	8	9	10	11	12	15	17	18	21	22	23	25	27	
**Ascomycota**																
*Scytalidium album*	95%	1	1			3	3	2		1	4		1	9	13	**38**
*Mariannaea camptospora*	100%		2	1			1		2		7			1	8	**22**
*Hypocrea pachybasioides*	99%	1	1	3	3	3	3			2	3				1	**20**
*Pseudogymnoascus sp*. *E*	99%	1	1	3	3	9		1								**18**
*Oidiodendron griseum*	99%		1		1		3							6	4	**15**
*Pochonia bulbillosa*	99%		1								5			2	5	**13**
*Penicillium spinulosum*	99%			1		1	4	2			4					**12**
*Cosmospora viridescens*	99%	1	3		4									2		**10**
*Penicillium montanense*	99%	1	2	1	1						3		1			**9**
*Penicillium ubiquetum*	100%				1	3	2		1	1	1					**9**
*Oidiodendron truncatum*	99%		2	1				1						3		**7**
*Penicillium raphiae*	99%	1			1									3	2	**7**
*Scytalidium circinatum*	95%					1									5	**6**
*Penicillium echinulatum*	99%				1		1						1	3		**6**
*Calcarisporium arbuscula*	98%			1	1						3					**5**
*Calcarisporium cordycipiticola*	92%						2								3	**5**
*Oidiodendron truncatum*	96%		1	1		1									2	**5**
*Trichoderma oblongisporum*	99%			1							2			1	1	**5**
**Basidiomycota**																
*Postia floriformis*	94%		2		7	1	3	2	2	1	4				4	**26**
*Sistotrema brinkmannii*	100%		3	2	1		5				4	1		10		**26**
*Calocera cornea*	87%		9		4	1	1			1	4			1	3	**24**
*Hypochnicellum molle*^1^	100%						3	1	1					1	5	**11**
*Postia floriformis*	99%					1	6								2	**9**
*Armillaria sinapina*	99%	4			2		1									**7**
*Hyphodontia floccosa*	99%			2	1				1						1	**5**
*Jaapia argillacea*	98%						1				4					**5**
**Mucoromycota**																
*Mortierella parvispora*	98%			1	2	3	3	1	3		5		2	2	5	**27**
*Mortierella cf*. *gamsii*	98%		8	2	2	1			1							**14**
*Mortierella hyalina*	99%	1	4	2			1				3				1	**12**
*Mortierella basiparvispora*	99%		2	1	3				1						2	**9**
*Mortierella hyalina*	93%			1	1						7					**9**
*Mucor heimalis*	100%	1	1				2				2					**6**
*Mortierella gemmifera*	99%		1	1	1						2					**5**
*Mortierella parvispora*	96%										4				1	**5**
*Mortierella pulchella*	99%		1		2						1			1		**5**
**# of Samples**		8	27	16	34	18	58	14	9	11	60	1	3	37	67	

^1^Based on large subunit (LSU) sequence

**Fig 1:**
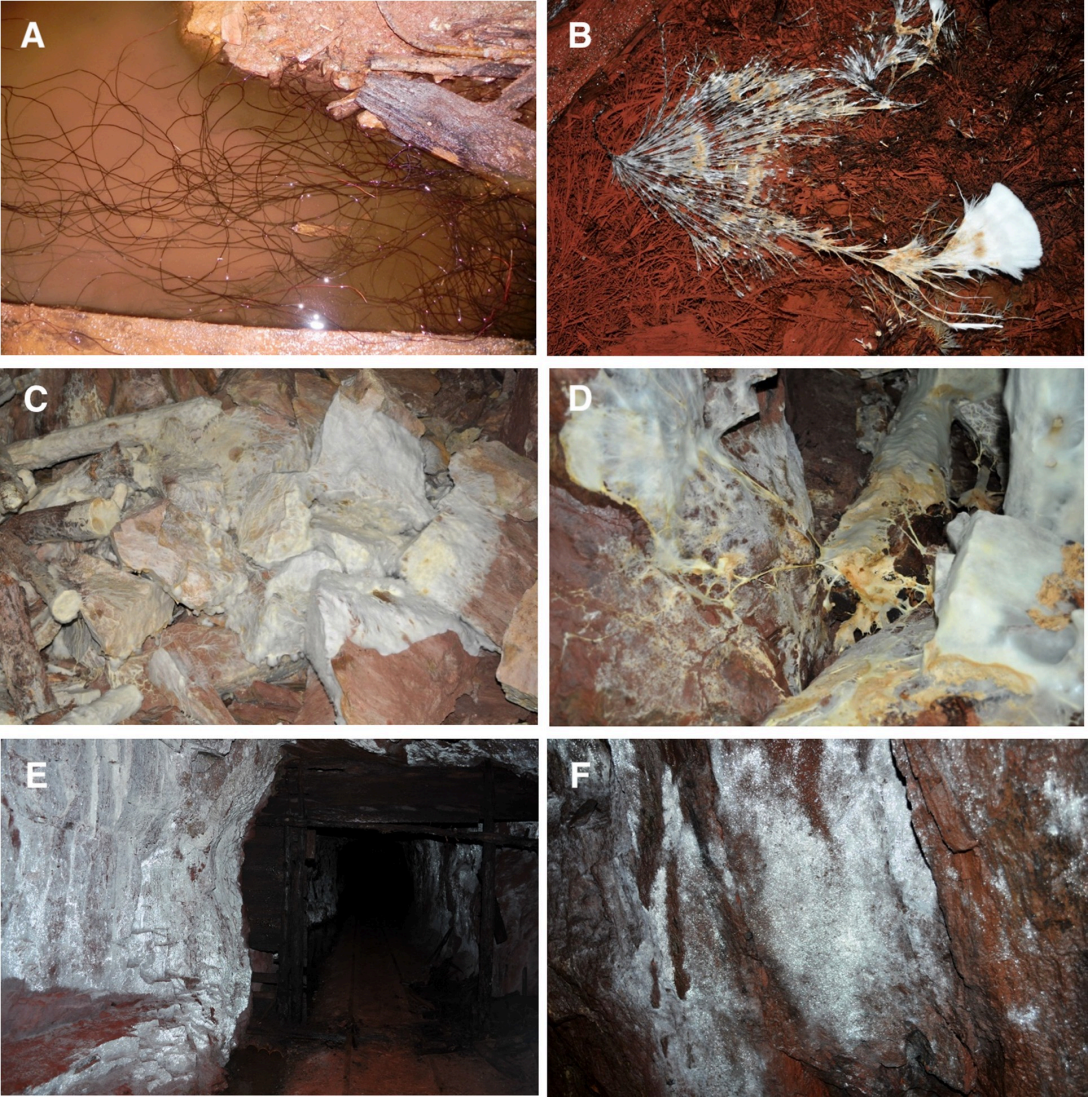
Images of unusual fungal growth in the Soudan mine. (A) masses of rhizomorphs of *Armillaria sinapina* growing on logs and in a water pool. (B) fresh mycelial growth of *A*. *sinapina* on the surface of a water pool with large amounts of rhizomorphs on the bottom of the pool heavily laden with iron and other metals. (C) large area of fungal mycelium of *Postia* sp. growing from wood material over rock surfaces. (D) *Postia* sp. forming rhizomorphs from wood growth enabling it to transport nutrient to the advancing front as it grows across rock surfaces. (E) growth of *Pseudogymnoascus* sp. over large areas of wall and ceiling. (F) *Pseudogymnoascus* sp. growing on the rock surface of the mine wall.

Unusual growth forms of several fungi were observed. Black rhizomorphs, abundant in many locations throughout the mine, were cultured and identified as *Armillaria sinapina*. They were present in large quantities on levels 10 and 27 growing in pools of water and on the floor of the mine. The rhizomorphs grew on wood and also extended out on the floor of the mine. In some areas the rhizomorphs were growing long distances between wood substrates (20+ ft). Also pools of water between wood sources had masses of rhizomorphs that appeared to have accumulated over many decades of growth (Fig 1). Large masses of white mycelial fans were also found on other timbers, and identified as *Postia* sp. On level 25, a large expanse of mycelium was growing on mine timbers from a small collapsed side stope and extended out over boulders for several meters (Fig 1). Large white rhizomorphs were also observed among the mycelial growth on wood, collapsed rocks and rock walls. Also, at another location (level 12), a pile of lumber left in the mine was completely covered in mycelium, which did not appear to be actively growing. Several fungi were cultured from the mycelium, however, and macroscopic characteristics of the growth form were similar to the *Postia* sp. on level 25. Expansive white fungal growth was also observed on the walls and ceiling of several levels. The coverage of growth in these areas varied between several m^2^ to very large areas of the walls and ceiling (Fig 1). Fungi grown from swabs taken from these areas showed the dominant fungi isolated were *Pseudogymnoascus* species. Phylogenetic analyses showed several different species of *Pseudogymnoascus* were present and this information has been previously published [26].

**Fig 2:**
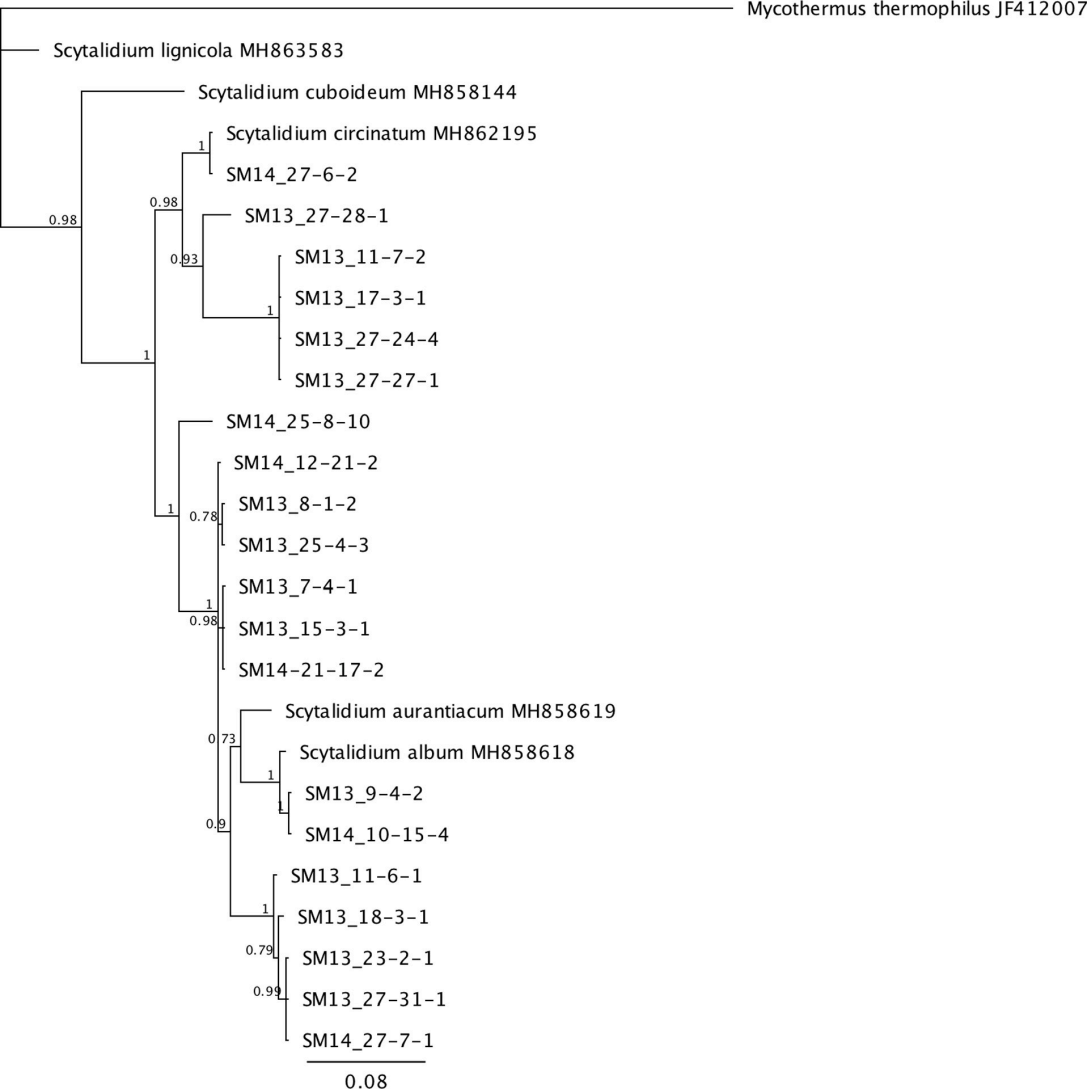
Bayesian tree constructed using ITS sequences from *Scytalidium* species isolated from the Soudan mine (preceded with “SM”) and related genera. Posterior probabilities are shown at branches.

**Fig 3:**
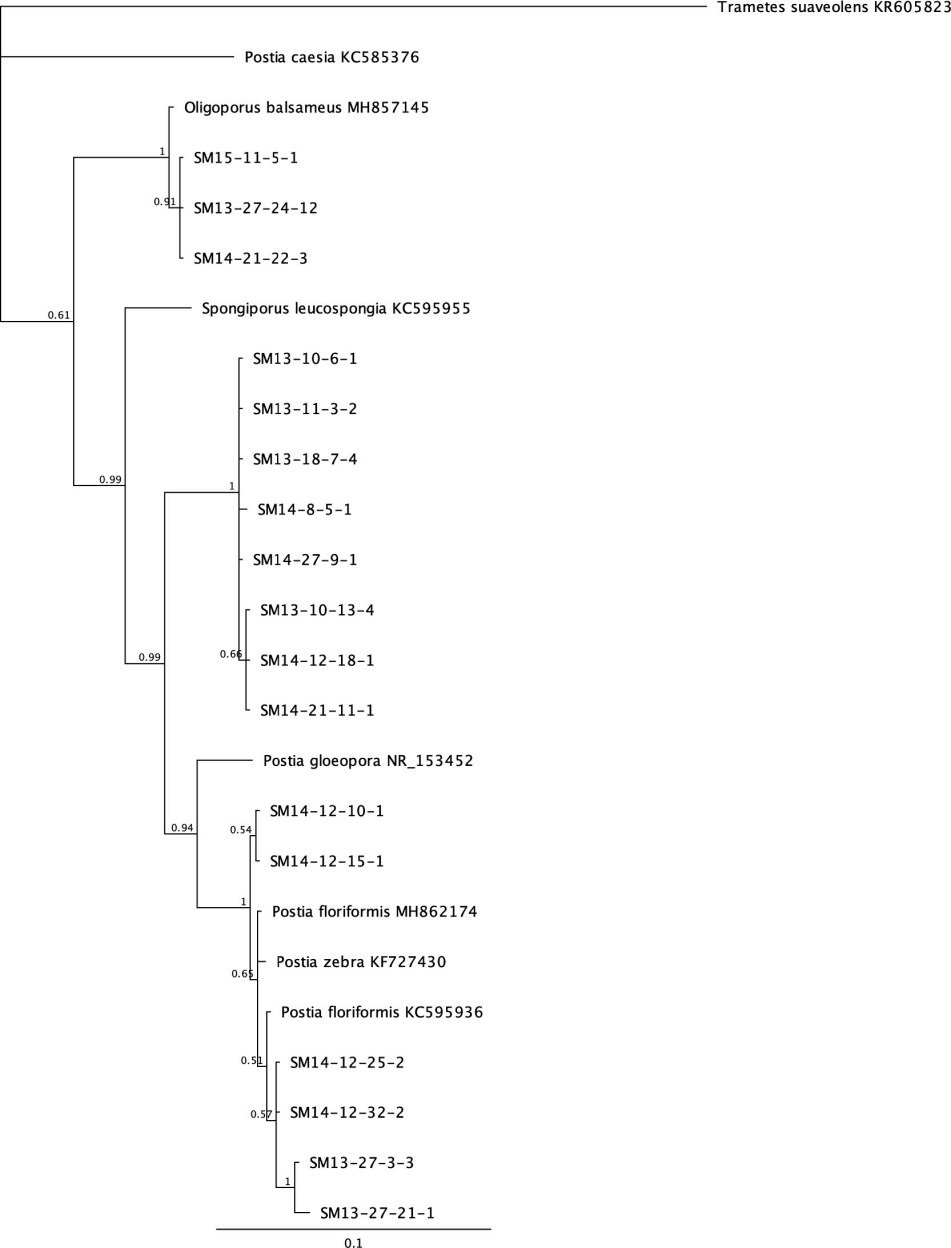
Bayesian tree constructed using ITS sequences from *Postia* species isolated from the Soudan mine (preceded with “SM”) and related genera. Posterior probabilities are shown at branches.

**Fig 4:**
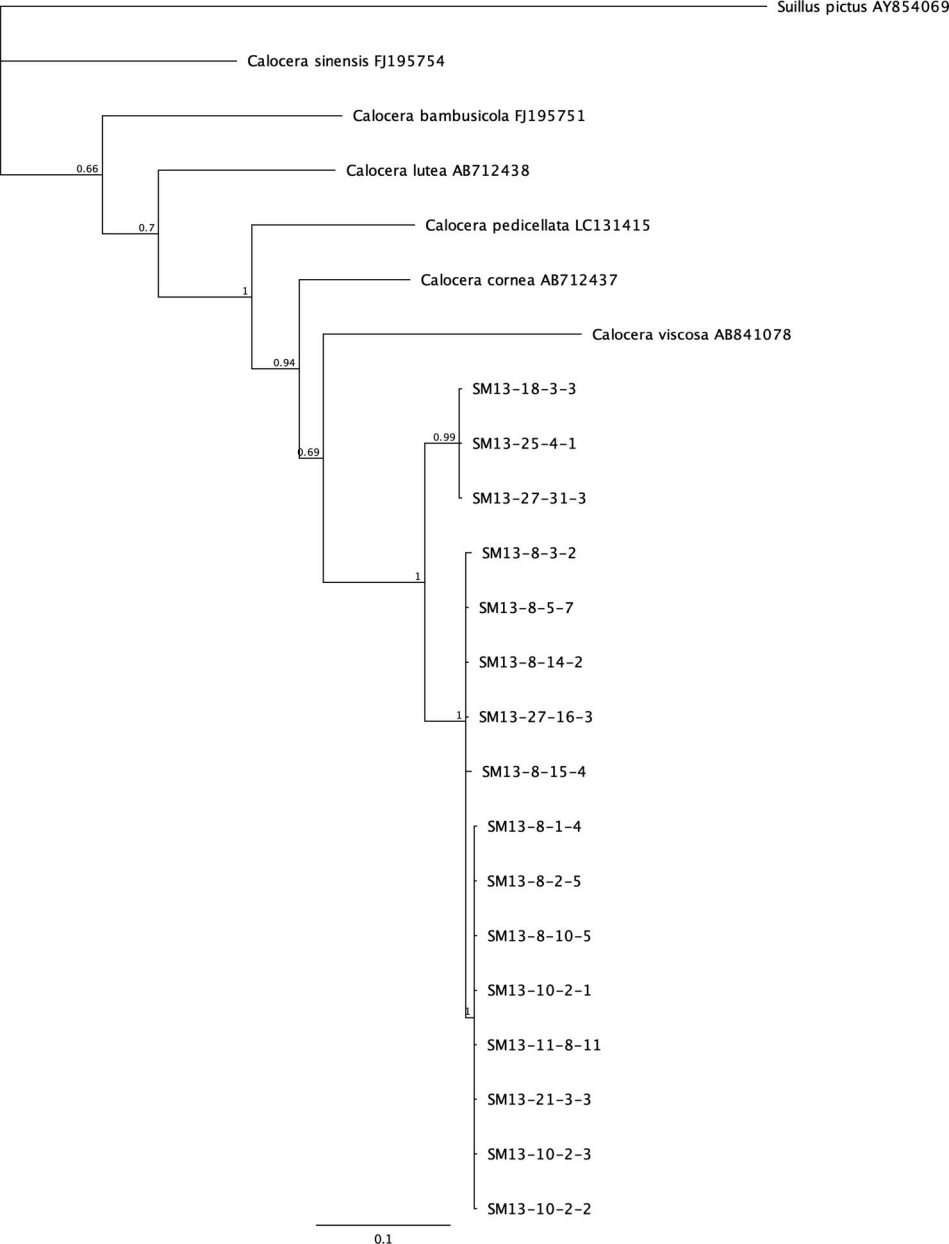
Bayesian tree constructed using ITS sequences from *Calocera* species isolated from the Soudan mine (preceded with “SM”) and related genera. Posterior probabilities are shown at branches.

Phylogenetic analysis was done on the most prevalent fungi that appeared to be undescribed species from BLAST search results. *Scytalidium* spp. isolated from 13 levels of the mine consist of a complex of species with most isolates representing undescribed species (Fig 2). Species fall into two main clades, one mostly related to *S*. *circinatum* and one closest to *S*. *album* and *S*. *aurantiacum*. The majority of the *Postia* species encountered (26 of 35) are in a well supported separate clade not shared with a described species, while additional isolates share the *Postia floriformus* / *P*. *zebra* clade (Fig 3). Three additional isolates group in a well supported sister clade to that of *Oligoporus balsameus*. A recent study on the phylogeny and taxonomy of *Postia* proposed changes in the naming of several closely related groups based on molecular and taxonomic data [27]. The *Postia* sp. studied here (with the exception of those closest to *O*. *balsameus*) would be considered *Spongiporus* instead of *Postia*, but for the purposes of this study we are keeping the genus name that is used with GenBank reference sequences. The isolates of *Calocera* represent undescribed species that are most closely related to *C*. *viscosa*. Three distinct well supported clades of these fungi were evident, however, branches to related species *C*. *viscosa*, *C*. *cornea* and *C*. *pendicellata* were less supported (Fig 4.)

**Fig 5:**
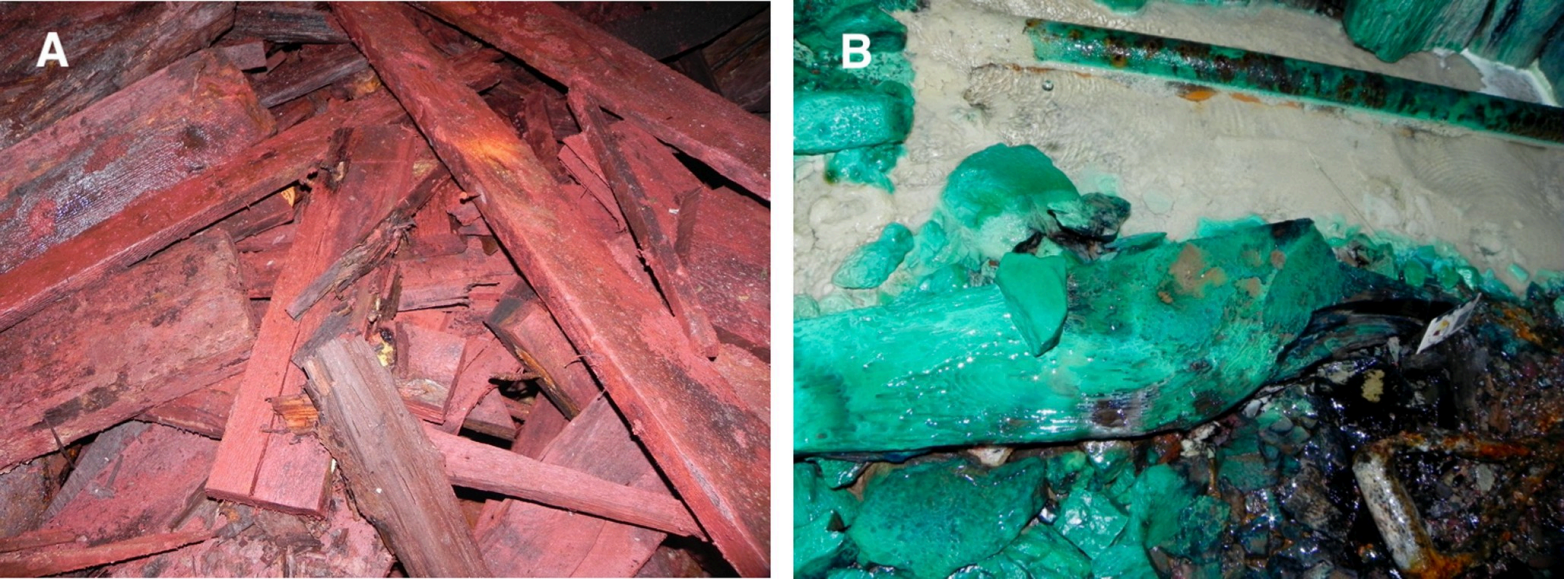
Examples of extensive metal deposition on wood in the Soudan mine. (A) A pile of discarded lumber coated with iron and (B) encased with a layer of copper and despite the extremely high metal concentrations, fungi such as *Cadophora* species were isolated from these substrates.

The elemental analysis of samples from 12 different levels revealed remarkably high concentrations of metal ions present (Table 2) in all wood samples and *Armillaria* rhizomorphs from the mine (Fig 5). Some of the highest concentrations (ppm) were 65,867 Al, 44,729 Ca, 1179 Co, 5843 Cu, 26,591 Fe, 26,490 K, 14,207 Mg, 15,725 Mn, 13,608 Na, 817 Ni, 1599 Pb and 1847 Zn.

**Table 2:** Elemental analysis (ppm) of woods and *Armillaria* rhizomorphs collected from various levels of the Soudan iron ore mine and control sound wood used for comparison.

Mine Level- sample #	Al	B	Ca	Cd	Co	Cr	Cu	Fe	K	Mg	Mn	Mo	Na	Ni	Pb	Zn	P
**7–1 rhizomorphs**	2821	15	28215	4	5	9	144	9741	10435	3127	359	0	621	46	44	1319	745
**8–2 wood**	1776	34	22679	1	11	6	159	9557	1023	3056	639	1	629	41	28	313	901
**8–15 wood**	324	16	11856	1	3	1	43	1518	356	1622	152	0	427	10	13	317	536
**10–10 wood**	65867	29	2664	0	1179	9	5843	2447	345	243	15725	0	170	817	2	132	3903
**10–14 rhizomorphs**	313	6	32040	1	7	2	121	350	13895	3049	1756	0	403	325	2	376	877
**11–7 wood**	56	11	7862	1	6	1	8	97	848	1653	680	0	68	3	1	60	81
**11–8 wood**	2692	8	19190	0	3	1	355	840	57	2338	88	2	458	11	1	15	372
**12–6 wood**	166	17	7770	0	1	1	30	6533	117	2517	158	0	325	4	1599	1223	107
**12–8 wood**	1840	26	6288	1	5	4	178	9940	566	1019	691	0	885	25	22	54	274
**12–12 wood**	254	5	7250	1	13	1	29	1126	< 0.30	1529	281	0	44	28	3	62	188
**15–1 wood**	432	6	3541	0	1	1	181	655	745	751	167	0	983	2	4	53	252
**15–6 wood**	1313	12	23814	1	2	18	92	2891	5334	3672	79	0	1193	6	27	350	297
**17–2 wood**	90	17	10984	0	0	1	87	153	195	5048	844	0	1787	1	320	197	160
**18–2 wood**	1406	10	6426	0	10	3	20	2159	177	2727	431	0	366	14	7	203	233
**21–17 wood**	305	26	745	0	2	1	9	12932	< 0.30	471	13	0	173	1	4	6	208
**21–19 wood**	2351	10	3375	0	9	2	28	2740	829	1497	97	0	358	41	9	44	164
**21–20 wood**	247	9	8053	11	1	2	56	1861	26490	3793	74	0	4564	2	15	179	6426
**21–22 wood**	89	15	19396	1	0	1	7	457	6168	2729	286	0	3576	1	3	86	141
**23–1 wood**	1815	66	44729	1	10	26	393	16485	2760	14207	581	0	10144	44	131	1847	769
**25–4 wood**	188	57	4812	1	1	1	10	21850	2765	1068	112	0	11279	1	3	27	129
**27–24 wood**	917	15	19873	1	4	4	39	2998	1484	1403	108	0	2651	7	7	381	370
**27–31 wood**	799	59	38912	0	1	3	119	26591	734	2169	40	0	13608	1	10	8	312
**27–35 wood**	1191	27	14520	2	5	20	94	10116	26	2484	251	0	1949	9	40	1433	159
**27–43 wood**	121	16	< 0.43	0	1	1	53	1160	234	5136	171	0	9369	1	2	56	150
**Pine wood control**	8	2	426	0	0	1	2	12	886	124	45	0	13	1	0	10	165
**Oak wood control**	1	3	1125	0	0	0	1	5	405	6	11	0	5	1	0	1	5

## Discussion

This study provides new information on the fungal diversity of an understudied iron ore mine environment, and revealed a diverse assemblage of taxa. Several conspicuous and unusual growth forms of Basidiomycota were found and appear similar to those observed in some of the oldest references to subterranean fungi. First, *A*. *sinapina* rhizomorphs were observed in astounding quantities in several areas in the Soudan mine growing over long distances (>5m) between suitable timber substrates (Fig 1). Although the rhizomorphs were observed growing in many areas of the mine, isolation frequency of *A*. *sinapina* does not reflect its abundance because once rhizomorphs were identified they were not continually isolated. The rhizomorphic growth habit of *A*. *sinapina* in the mine is extraordinary, since it usually cannot be observed in its entirety when growing in its natural underground habitat, such as in tree roots and through soil. In forest disease situations, the fungus uses this highly melanized growth form to spread through the soil from host to host and over time the fungus can move across vast distances [28]. Melanin production in fungi have an array of biological roles and act as binding sites for metals [29–31], and *Armillaria* rhizomorphs have been shown to absorb large concentrations of metal ions in soils and have been suggested to help protect the rhizomorphs from other antagonistic fungi [31]. Despite the high concentrations of heavy metals in the mine water [15] and exceedingly high concentrations that accumulate in the wood (Table 2), *A*. *sinapina* has been able to tolerate these conditions and grow prolifically as it seeks out new woody substrates. There was also a similar observation of *Armillaria* rhizomorphs in an abandoned copper mine in Michigan [5].

*Postia* was the most frequently isolated Basidiomycota in the mine. It was distributed throughout the mine and was isolated from 9 of the 14 levels that were sampled. Conspicuous mycelial fans and mats were observed as well as white mycelial cords that grew over wood and rock surfaces (Fig 1). These structures are similar to the growth habit of *Serpula* species and a few other decay fungi that are common in wooden building environments [32]. The production of thick white rhizomorphs enable these fungi to grow over non-nutrient surfaces until they encounter a suitable substrate. These *Postia* sp. appear well adapted to the underground mine environment producing white rhizomorphs to move across mineral surfaces and to protect it from competing organisms. The phylogenetic analysis (Fig 3) show diverse species in this group and the majority of *Postia* isolates from the mine appear to be undescribed species.

Another unusual discovery is the presence of several Basidiomycota, *Hypochnicellum*, *Jaapia* and *Sistotrema*, which have also been found to occur in extreme polar environments and in wooden buildings in cold regions of the world. As part of the corticioid group, these species have resupinate fruiting structures that are effuse and spreading, often forming on the underside of logs. Most species are wood degraders. *Hypochnicellum* and *Jaapia* have been isolated from Arctic driftwood as well as historic wooden structures located in Antarctica [16,33,34]. *Jaapia* is a relatively rare genus but has a wide geographical distribution [35] and accommodates two species; *J*. *argillacea* and *J*. *ochroleuca*. *Sistotrema brinkmannii* has been identified in soils and wood from Antarctica [34,36] and has also been frequently isolated from coal mines in the United States [6]. This species has a wide host range and has been associated with the wood of angiosperms and gymnosperms, soil, moss and reported to cause a brown rot in wood [37,38]. The ITS BLAST results from *Hypochnicellum* isolates resulted in a low match to *Amylothelia crassicula*, however, sequences obtained from the large subunit (LSU) and queries of other databases (CBS and UNITE) show a 100% match to *H*. *molle*. Unfortunately, an ITS sequence of a documented *H*. *molle* has not been accessioned in GenBank. This fungus has several references to polar or alpine environments and has been reported to cause decay in buildings in Norway and Germany [39–41]. One likely adaptation mechanism of *Sistotrema* and *Hypochnicellium* to extreme environments is the production of basidiospores and chlamydospore-like structures, respectively, produced from the mycelium which may aid in dispersal and are resistant to adverse conditions. The abundance of these Basidiomycota in the mine and other extreme environments suggests they may have an important ecological role for recycling carbon.

*Calocera* sp. was also a dominant basidiomycete that was isolated from the mine. This fungus belongs in the Dacrymycetes or one of the ‘jelly fungi’. The closest GenBank match using BLAST was 87% to *C*. *cornea*. Phylogenetic analysis shows this is a new species of *Calocera* (Fig 4). *C*. *cornea* produces yellow, tapered, finger-like basidiocarps, which were not observed in the mine. It is often referenced as being associated with decayed wood, and produces a brown pocket rot [42].

Ascomycota was the major group of fungi that were represented in the Soudan mine. Our findings are similar to other studies of fungi in subterranean ecosystems such as caves and mines where Ascomycota are common [8]. However, among the Ascomycota that we isolated, 26 taxa do not match described species (ie <97% BLAST match). The mine contained many unique fungal species and several undescribed species that need additional study. A *Scytalidium* species that matched 95% to *S*. *album* was the most often isolated of the Ascomycota. However, phylogenetic analysis of all *Scytalidium* sp. isolated shows considerable genetic variability in with several clades comprising undescribed species (Fig 2). *Scytalidium* sp. have adaptive characteristics that may aid in their abundance in the mine. Previous studies have shown varied antagonistic properties from *Scytalidium* spp. Compounds isolated and purified from *Scytalidium album* have shown antifungal, antimicrobial and anticancer properties [43] including the antibiotic scytalidin [44]. Several species including *S*. *album* have also been described as mycoparasites, parasitizing a wide range of Basidiomycota [45–47] and have been suggested for potential use in biocontrol of wood decay and other fungi [48]. Other species such as *S*. *circinatum* and *S*. *lignicola* have been associated with the colonization and decay of utility poles, indicating a tolerance to the harsh preservatives creosote and pentachlorophenol used in the poles [49,50]. The antagonistic characteristics of *Scytalidium*, along with its ability to colonize and degrade wood show adaptive traits enabling these species to grow in adverse conditions and dominate fungal communities in the mine.

Compared with other fungi, entomopathogens are the most studied in underground environments [8]. While insects were not a target for fungal isolations in these investigations, several important genera of insect associated fungi were identified. *Mariannaea camptospora*, the second most isolated Ascomycota has been previously described as a pathogen of insects [51], isolated from decaying wood [52]. Other insect related fungi that were identified include three *Lecanicillium* species which are closely related anamorphs of *Cordyceps* and *Tolypocladium*. *Pochonia* sp. are nematophagous fungi that are commonly found in soils [53]. Very little is known about subterranean nematodes in mines and caves and their ecology and natural history needs additional study [54].

*Pseudogymnoascus* species (formerly *Geomyces*) found covering large areas of rock on mine walls and ceilings indicates a unique adaption and ecological function in subterranean environments. Comparisons of ITS sequences using BLAST showed matches to similar taxa isolated from cave or mine hibernacula, but not matching described species of *Pseudogymnoascus*. This group is related to the white -nose syndrome causal agent, *P*. *destructans* [26]. While many *Pseudogymnoascus* species are often associated with terrestrial soils and they would be expected to be isolated from subterranean environments, the extent of their growth within the mine was extraordinarily high. While fungi associated with rock surfaces of subterranean and above ground environments [12,55,56] have had some study, it is not clear what nutrient sources are being utilized by these species. We hypothesize that because many of the areas where growth is occurring on the walls and ceiling are wet, moisture may be leaching additional nutrients to wall and ceiling surfaces. Ogórek et al. 2017 [57] also reports *Geomyces* (*Pseudogymnoascus*) *pannorum* in high abundance of the walls of an aluminous shale mine in Poland. Biolog data from [26] also showed that the *Pseudogymnoascus* spp. isolated from the mine have broad enzyme capabilities which are similar to their *Geomyces* counterparts commonly found in terrestrial soils.

*Mortierella* species are cosmopolitan fungi that are frequently recovered from soils where they maintain a saprobic lifestyle. Many species are capable of metabolizing chitin [58], are commonly found in the subterranean environments [59], show metal tolerance [60], and have psychrotolerance [61]. In addition, many species are well known for producing industrially relevant fatty acids [62]. Chitin is a component of insectivorous bat guano and may be a nutrient source for some of these species in or near bat hibernacula [63]. The 24 *Mortierella* taxa found in the mine, while unexpected, may be reflective of the diversity in the genus *Mortierella*, containing nearly 100 species [64]. Of the 31 taxa we found in this study, 9 lacked ITS sequence similarity to described species indicating the need for species descriptions. *Mortierella parvispora* was particularly abundant compared to others species and was encountered nearly twice that of the next most isolated species. A previous study showed that cell walls of *M*. *parvispora* are resistant to microbial enzyme digestion, and the authors suggest that the mechanisms involved in protection from other organisms may also protect them from the extreme mine environment [65].

Water that is continually pumped from the Soudan mine is contaminated with metal ions [15]. The baseline concentrations of metals in mine water have been shown to be quite low compared to wood samples tested (Table 2). In the water, concentrations of copper (Cu) ranges from 0.083–0.5 ppm, cobalt (Co) 0.006–0.026 ppm and mercury (Hg) 0.0004–0.00006 ppm [66] and in wood samples concentrations were highly elevated ranging from 7 to 5842 (Cu) and 1 to 1178 (Co) (Hg in wood was not measured). Wood from many areas in the mine have exceedingly high concentrations of metals and despite this, fungi are present and have been isolated from these samples. Level 10 has an area where very high amounts of copper have leached into a standing pool of water, and has stained adjacent rocks, parts of the wall and pieces of wood green. In particular, samples from the 10^th^ level had very high copper, cobalt and aluminum concentrations. Among the fungi isolated from samples from this area of the mine was *Cadophora fastigiata*. Species in this genus have highly melanized hyphae and have been identified in many extreme environments like heavy metal contaminated sites [67], polar environments [16,34,67,68] deep sea hydrothermal vents [69], saline and acidic soils [70] and copper treated wood [71]. In a previous study of secondary metabolites from fungi, several new isochromanone compounds (“soudanones”) with activity against *Cryptococcus* and *Candida* pathogens were characterized from a culture of *Cadophora fastigiata* that was isolated from wood with high copper from level 10 in the mine [72]. The presence of *Cadophora* species in these extreme environments indicates an adaptation to withstand high heavy metals and salts, cold temperature and other stress factors. Wood in this mine environment appears to act as a metal ion sink where these metals accumulate over time resulting in much higher concentrations than is found in water from the mine. This also illustrates that fungal species isolated from extreme environments are often targets for the novel compounds these organisms produce to adapt in the harsh environment.

Mines, unlike other subterranean environments such as caves, are relatively recent anthropogenic disturbances. Consequently, fungi and other organisms have been introduced by a variety of modes and the environment selects for those species that are able to adapt [73]. The mine can be a harsh environment, however, with high concentrations of metal ions and high salt concentrations compared to the surface. The thousands of wood timbers in the Soudan mine also provide a nutrient source for microbes that is normally not found in oligotrophic caves. The obvious and main sources of introduction of these fungi were with the wood that has been brought into the mine during operations as well as air exchange, infiltration of rain and ground water, and human, animal, and insect activity. In the Ascomycota assemblage, there were 88 taxa that were recovered two or fewer times suggesting many of these taxa are not common species and may enter the mine as spores introduced by air currents or other avenues. It would be expected that certain selection pressures (limited light, cool temperatures, high metal concentrations) will be conducive to growth of some fungi that are well adapted to these conditions and would inhibit or limit colonization by others. In addition, timbers, especially large amounts of wood found in some areas of the Soudan mine, result in fungal hotspots compared to more oligotrophic areas of the mine. While temperature does not vary widely from level to level, the amount of moisture varies significantly. On several levels, excess water has collected and is pumped to the surface to avoid flooding and in these areas wood is very wet compared to other areas of the mine where very little water is present. The results presented are consistent with other studies of subterranean environments and many fungi inside the mine broadly reflect the generic diversity found in the above ground environment [8]. However, many fungi isolated from the Soudan mine revealed sufficient sequence differences to suggest there are new species to be described from this subterranean environment. This work provides new information to the growing body of studies on subterranean mycology. These fungi provide a rich resource for exploration of diverse applications. Studies are underway to evaluate them for metal sequestration, biocontrol for WNS and new drug discovery. Future exploratory work using amplicon sequencing of not only fungi but also bacteria would be useful in characterizing the mine microbiomes and relationships among these organisms.

## Supporting information

S1 Table Table showing best BLAST % identity, numbers of isolates recovered from each mine level, and GenBank # for each taxa. (DOCX)
