# Supplementary material for: Diverse subterranean fungi of an underground iron ore mine
Source: PLoS One. 2020 Jun 4;15(6):e0234208. doi: 10.1371/journal.pone.0234208 (PMC7272026; doi:10.1371/journal.pone.0234208)
Supplement: S1 Table — (DOCX) [file pone.0234208.s001.docx]

S1 Table. Table showing best BLAST % identity, numbers of isolates recovered from each mine level, and GenBank # for each taxa.

| **Fungal Taxa** | **%**  **Identity** | **Level** | | | | | | | | | | | | | | **Total** | **Genbank #** |
| --- | --- | --- | --- | --- | --- | --- | --- | --- | --- | --- | --- | --- | --- | --- | --- | --- | --- |
|  |  | **7** | **8** | **9** | **10** | **11** | **12** | **15** | **17** | **18** | **21** | **22** | **23** | **25** | **27** |  |  |

| **Ascomycota** | | | | | | | | | | | | | | | | | |
| --- | --- | --- | --- | --- | --- | --- | --- | --- | --- | --- | --- | --- | --- | --- | --- | --- | --- |
| *Scytalidium album* | 95% | 1 | 1 |  |  | 3 | 3 | 2 |  | 1 | 4 |  | 1 | 9 | 13 | 38 | MN905769 |
| *Mariannaea camptospora* | 100% |  | 2 | 1 |  |  | 1 |  | 2 |  | 7 |  |  | 1 | 8 | 22 | MN905770 |
| *Hypocrea pachybasioides* | 99% | 1 | 1 | 3 | 3 | 3 | 3 |  |  | 2 | 3 |  |  |  | 1 | 20 | MN905771 |
| *Pseudogymnoascus sp. E* | 99% | 1 | 1 | 3 | 3 | 9 |  | 1 |  |  |  |  |  |  |  | 18 | KY270902 |
| *Oidiodendron griseum* | 99% |  | 1 |  | 1 |  | 3 |  |  |  |  |  |  | 6 | 4 | 15 | MN905772 |
| *Pochonia bulbillosa* | 99% |  | 1 |  |  |  |  |  |  |  | 5 |  |  | 2 | 5 | 13 | MN905773 |
| *Penicillium spinulosum* | 99% |  |  | 1 |  | 1 | 4 | 2 |  |  | 4 |  |  |  |  | 12 | MN905774 |
| *Cosmospora viridescens* | 99% | 1 | 3 |  | 4 |  |  |  |  |  |  |  |  | 2 |  | 10 | MN905775 |
| *Penicillium montanense* | 99% | 1 | 2 | 1 | 1 |  |  |  |  |  | 3 |  | 1 |  |  | 9 | MN905776 |
| *Penicillium ubiquetum* | 100% |  |  |  | 1 | 3 | 2 |  | 1 | 1 | 1 |  |  |  |  | 9 | MN905777 |
| *Oidiodendron truncatum* | 99% |  | 2 | 1 |  |  |  | 1 |  |  |  |  |  | 3 |  | 7 | MN905778 |
| *Penicillium raphiae* | 99% | 1 |  |  | 1 |  |  |  |  |  |  |  |  | 3 | 2 | 7 | MN905779 |
| *Scytalidium circinatum* | 95% |  |  |  |  | 1 |  |  |  |  |  |  |  |  | 5 | 6 | MN905932 |
| *Penicillium echinulatum* | 99% |  |  |  | 1 |  | 1 |  |  |  |  |  | 1 | 3 |  | 6 | MN905780 |
| *Calcarisporium arbuscula* | 98% |  |  | 1 | 1 |  |  |  |  |  | 3 |  |  |  |  | 5 | MN905781 |
| *Calcarisporium cordycipiticola* | 92% |  |  |  |  |  | 2 |  |  |  |  |  |  |  | 3 | 5 | MN905782 |
| *Oidiodendron truncatum* | 96% |  | 1 | 1 |  | 1 |  |  |  |  |  |  |  |  | 2 | 5 | MN905783 |
| *Trichoderma oblongisporum* | 99% |  |  | 1 |  |  |  |  |  |  | 2 |  |  | 1 | 1 | 5 | MN905784 |
| *Cadophora melinii* | 99% |  | 2 |  | 1 |  |  |  |  |  |  |  |  |  | 1 | 4 | MN905785 |
| *Hypocrea koningii* | 100% |  | 1 |  |  |  |  |  |  |  | 3 |  |  |  |  | 4 | MN905786 |
| *Lecanicillium fusisporum* | 93% |  |  |  |  |  |  |  |  |  | 2 |  |  |  | 2 | 4 | MN905787 |
| *Lecanicillium primulinum* | 99% |  |  |  |  |  |  |  |  |  |  |  |  |  | 4 | 4 | MN905788 |
| *Lecanicillium psalliotae* | 99% |  |  |  |  |  |  |  |  |  |  |  |  |  | 4 | 4 | MN905789 |
| *Patinella hyalophaea* | 100% |  |  |  | 2 | 2 |  |  |  |  |  |  |  |  |  | 4 | MN905790 |
| *Tolypocladium inflatum* | 100% |  |  |  | 2 | 2 |  |  |  |  |  |  |  |  |  | 4 | MN905791 |
| *Allantonectria miltina* | 92% |  | 1 |  | 1 |  |  | 1 |  |  |  |  |  |  |  | 3 | MN905792 |

| **Fungal Taxa** | **%**  **Identity** | **Level** | | | | | | | | | | | | | | **Total** | **Genbank #** |
| --- | --- | --- | --- | --- | --- | --- | --- | --- | --- | --- | --- | --- | --- | --- | --- | --- | --- |
|  |  | **7** | **8** | **9** | **10** | **11** | **12** | **15** | **17** | **18** | **21** | **22** | **23** | **25** | **27** |  |  |

| *Apiotrichum dulcitum* | 100% |  | 1 |  | 1 |  | 1 |  |  |  |  |  |  |  |  | 3 | MN905793 |
| --- | --- | --- | --- | --- | --- | --- | --- | --- | --- | --- | --- | --- | --- | --- | --- | --- | --- |
| *Coniochaeta mutabilis* | 100% |  | 3 |  |  |  |  |  |  |  |  |  |  |  |  | 3 | MN905794 |
| *Cyphellophora olivacea* | 99% |  |  |  |  |  |  |  |  | 1 |  |  |  | 1 | 1 | 3 | MN905795 |
| *Oidiodendron tenuissimum* | 98% |  |  |  |  |  |  |  |  |  | 2 |  |  |  | 1 | 3 | MN905796 |
| *Engyodontium album* | 99% |  |  |  |  |  |  |  | 1 |  |  |  | 1 | 1 |  | 3 | MN905797 |
| *Mammaria echinobotryoides* | 100% |  |  | 1 | 2 |  |  |  |  |  |  |  |  |  |  | 3 | MN905798 |
| *Penicillium chrysogenum* | 99% | 1 |  |  |  |  |  |  |  |  |  |  |  | 2 |  | 3 | MN905799 |
| *Penicillium sanguifluum* | 99% |  |  |  |  |  |  |  |  |  |  | 1 |  | 1 | 1 | 3 | MN905800 |
| *Pyrenochaeta ligni-putridi* | 88% |  | 2 |  |  |  | 1 |  |  |  |  |  |  |  |  | 3 | MN905801 |
| *Scytalidium album* | 99% |  |  | 1 | 1 |  |  |  |  |  |  |  |  |  | 1 | 3 | MN905802 |
| *Cadophora malorum* | 99% |  |  |  |  |  |  |  |  | 1 |  |  |  | 1 |  | 2 | MN905803 |
| *Cercophora sparsa* | 99% |  | 1 | 1 |  |  |  |  |  |  |  |  |  |  |  | 2 | MN905804 |
| *Cladophialophora chaetospira* | 87% |  |  |  |  |  |  |  |  |  |  |  |  |  | 2 | 2 | MN905805 |
| *Cladosporium pseudocladosporioides* | 100% |  | 1 | 1 |  |  |  |  |  |  |  |  |  |  |  | 2 | MN905806 |
| *Elaphocordyceps subsessilis* | 99% |  |  |  | 1 | 1 |  |  |  |  |  |  |  |  |  | 2 | MN905807 |
| *Engyodontium album* | 99% |  |  |  |  |  |  |  | 1 |  |  |  |  |  | 1 | 2 | MN905808 |
| *Exophiala angulospora* | 99% |  |  |  |  |  |  |  |  |  |  |  | 1 |  | 1 | 2 | MN905809 |
| *Meliniomyces variabilis* | 93% |  |  |  | 2 |  |  |  |  |  |  |  |  |  |  | 2 | MN905810 |
| *Nectria flavoviridis* | 99% |  |  | 1 | 1 |  |  |  |  |  |  |  |  |  |  | 2 | MN905811 |
| *Nectria lugdunensis* | 99% | 1 |  |  |  |  | 1 |  |  |  |  |  |  |  |  | 2 | MN905812 |
| *Neobulgaria alba* | 95% | 1 |  |  | 1 |  |  |  |  |  |  |  |  |  |  | 2 | MN905813 |
| *Neodevriesia lagerstroemiae* | 88% |  |  |  |  |  |  |  |  |  |  |  |  | 2 |  | 2 | MN905814 |
| *Penicillium aeneum* | 99% | 1 |  |  |  |  |  |  |  |  |  |  |  | 1 |  | 2 | MN905815 |
| *Penicillium ardesiacum* | 100% |  |  |  |  |  |  |  |  |  |  |  |  | 2 |  | 2 | MN905816 |
| *Penicillium commune* | 99% |  |  |  |  |  | 1 |  |  |  |  |  |  | 1 |  | 2 | MN905817 |
| *Penicillium obscurum* | 100% |  | 1 |  | 1 |  |  |  |  |  |  |  |  |  |  | 2 | MN905818 |
| *Penicillium robsamsonii* | 99% |  |  |  |  |  |  |  |  |  |  |  |  |  | 2 | 2 | MN905819 |

| **Fungal Taxa** | **%**  **Identity** | **Level** | | | | | | | | | | | | | | **Total** | **Genbank #** |
| --- | --- | --- | --- | --- | --- | --- | --- | --- | --- | --- | --- | --- | --- | --- | --- | --- | --- |
|  |  | **7** | **8** | **9** | **10** | **11** | **12** | **15** | **17** | **18** | **21** | **22** | **23** | **25** | **27** |  |  |

| *Penidiella kurandae* | 88% |  |  |  |  |  |  |  |  |  |  |  |  | 2 |  | 2 | MN905820 |
| --- | --- | --- | --- | --- | --- | --- | --- | --- | --- | --- | --- | --- | --- | --- | --- | --- | --- |
| *Phialemonium inflatum* | 99% |  |  |  |  |  |  |  |  | 1 |  |  |  |  | 1 | 2 | MN905821 |
| *Pseudeurotium bakeri* | 99% | 1 |  |  |  |  |  |  |  | 1 |  |  |  |  |  | 2 | MN905822 |
| *Scytalidium circinatum* | 99% |  |  |  |  |  |  |  |  |  |  |  |  |  | 2 | 2 | MN905823 |
| *Sporothrix inflata* | 99% |  |  |  |  |  |  |  |  |  | 1 |  |  |  | 1 | 2 | MN905824 |
| *Zalerion varium* | 100% |  |  |  | 2 |  |  |  |  |  |  |  |  |  |  | 2 | MN905825 |
| *Absidia repens* | 97% |  |  |  |  |  | 1 |  |  |  |  |  |  |  |  | 1 | MN905826 |
| *Acanthostigma perpusillum* | 97% |  |  |  | 1 |  |  |  |  |  |  |  |  |  |  | 1 | MN905827 |
| *Acrodontium simplex* | 88% |  |  |  |  |  |  |  |  |  |  |  |  |  | 1 | 1 | MN905828 |
| *Acidothrix acidophila* | 99% |  |  |  |  |  |  |  |  |  |  |  |  |  | 1 | 1 | MN905829 |
| *Acremonium furcatum* | 98% |  |  |  |  |  |  |  |  |  |  |  |  |  | 1 | 1 | MN905830 |
| *Allantonectria miltina* | 92% |  |  |  |  |  | 1 |  |  |  |  |  |  |  |  | 1 | MN905831 |
| *Apiotrichum dulcitum* | 99% | 1 |  |  |  |  |  |  |  |  |  |  |  |  |  | 1 | MN905832 |
| *Ascocoryne cylichnium* | 92% |  |  |  | 1 |  |  |  |  |  |  |  |  |  |  | 1 | MN905833 |
| *Aspergillus flavus* | 99% |  |  |  | 1 |  |  |  |  |  |  |  |  |  |  | 1 | MN905834 |
| *Atractium stilbaster* | 99% |  |  | 1 |  |  |  |  |  |  |  |  |  |  |  | 1 | MN905835 |
| *Cadophora fastigiata* | 100% |  |  |  | 1 |  |  |  |  |  |  |  |  |  |  | 1 | MN905836 |
| *Cadophora sp. 5R24-1* | 98% |  |  |  |  |  |  |  |  |  |  |  |  | 1 |  | 1 | MN905837 |
| *Calcarisporium arbuscula* | 92% |  |  |  |  |  |  | 1 |  |  |  |  |  |  |  | 1 | MN905838 |
| *Rhinocladiella atrovirens* | 99% |  |  |  |  |  |  |  |  |  |  |  |  | 1 |  | 1 | MN905839 |
| *Cephalotheca sulfurea* | 90% |  |  |  |  |  |  |  | 1 |  |  |  |  |  |  | 1 | MN905840 |
| *Chloridium virescens* | 98% |  |  |  |  |  |  |  |  |  |  |  |  |  | 1 | 1 | MN905841 |
| *Cladophialophora mycetomatis* | 95% |  |  |  |  |  |  |  |  |  |  |  |  |  | 1 | 1 | MN905842 |
| *Cladosporium tenuissimum* | 100% | 1 |  |  |  |  |  |  |  |  |  |  |  |  |  | 1 | MN905843 |
| *Cordana inaequalis* | 97% |  |  |  |  |  |  |  | 1 |  |  |  |  |  |  | 1 | MN905844 |
| *Cosmospora obscura* | 99% |  |  |  |  |  |  |  | 1 |  |  |  |  |  |  | 1 | MN905845 |
| *Doratomyces stemonitis* | 99% |  |  |  | 1 |  |  |  |  |  |  |  |  |  |  | 1 | MN905846 |

| **Fungal Taxa** | **%**  **Identity** | **Level** | | | | | | | | | | | | | | **Total** | **Genbank #** |
| --- | --- | --- | --- | --- | --- | --- | --- | --- | --- | --- | --- | --- | --- | --- | --- | --- | --- |
|  |  | **7** | **8** | **9** | **10** | **11** | **12** | **15** | **17** | **18** | **21** | **22** | **23** | **25** | **27** |  |  |

| *Hawksworthiomyces crousii* | 94% |  |  |  |  |  |  |  |  |  | 1 |  |  |  |  | 1 | MN905847 |
| --- | --- | --- | --- | --- | --- | --- | --- | --- | --- | --- | --- | --- | --- | --- | --- | --- | --- |
| *Helicocarpus griseus* | 83% |  |  |  |  |  |  |  |  |  |  |  |  | 1 |  | 1 | MN905848 |
| *Hyaloscypha aureliella* | 96% |  |  |  | 1 |  |  |  |  |  |  |  |  |  |  | 1 | MN905849 |
| *Hyaloscypha vitreola* | 98% |  |  |  | 1 |  |  |  |  |  |  |  |  |  |  | 1 | MN905850 |
| *Ijuhya corynospora* | 86% |  |  |  |  |  |  |  |  |  |  |  |  |  | 1 | 1 | MN905851 |
| *Pochonia chlamydosporia* | 98% |  |  |  |  | 1 |  |  |  |  |  |  |  |  |  | 1 | MN905852 |
| *Mollisia dextrinospora* | 99% |  |  |  |  |  | 1 |  |  |  |  |  |  |  |  | 1 | MN905853 |
| *Mycosphaerella parva* | 86% |  |  |  |  |  |  |  |  |  |  |  |  | 1 |  | 1 | MN905854 |
| *Rhizodiscina lignyota* | 89% |  |  |  |  |  |  |  |  |  |  |  |  | 1 |  | 1 | MN905855 |
| *Byssochlamys spectabilis* | 99% |  |  |  |  |  | 1 |  |  |  |  |  |  |  |  | 1 | MN905856 |
| *Penicillium aurantiacobrunneum* | 99% |  |  |  |  |  | 1 |  |  |  |  |  |  |  |  | 1 | MN905857 |
| *Penicillium brevicompactum* | 99% |  |  |  |  |  |  |  |  |  |  |  |  | 1 |  | 1 | MN905858 |
| *Penicillium verrucosum* | 99% |  |  |  |  |  |  |  |  |  |  |  |  | 1 |  | 1 | MN905859 |
| *Penicillium wollemiicola* | 99% |  |  |  |  |  |  |  |  | 1 |  |  |  |  |  | 1 | MN905860 |
| *Rhizoscyphus ericae* | 99% |  |  |  | 1 |  |  |  |  |  |  |  |  |  |  | 1 | MN905861 |
| *Sagenomella humicola* | 84% |  |  |  |  |  |  |  |  |  |  |  |  | 1 |  | 1 | MN905862 |
| *Talaromyces atricola* | 99% |  |  |  |  |  |  |  |  |  |  |  |  |  | 1 | 1 | MN905863 |
| *Trichocladium opacum* | 99% |  |  |  | 1 |  |  |  |  |  |  |  |  |  |  | 1 | MN905864 |
| *Trichoderma appalachiense* | 100% |  |  |  |  |  | 1 |  |  |  |  |  |  |  |  | 1 | MN905865 |
| *Trichoderma atroviride* | 100% |  |  |  |  |  |  |  |  |  | 1 |  |  |  |  | 1 | MN905866 |
| *Trichoderma fomiticola* | 99% |  |  |  |  |  |  |  |  |  | 1 |  |  |  |  | 1 | MN905867 |
| *Trichoderma parapiluliferum* | 99% |  |  |  |  |  |  |  |  |  | 1 |  |  |  |  | 1 | MN905868 |
| *Trichoderma piluliferum* | 99% |  |  |  |  |  |  |  |  |  | 1 |  |  |  |  | 1 | MN905869 |
| *Trichoderma piluliferum* | 99% |  |  |  |  |  |  |  |  |  | 1 |  |  |  |  | 1 | MN905870 |
| *Trichoderma simmonsii* | 100% |  | 1 |  |  |  |  |  |  |  |  |  |  |  |  | 1 | MN905871 |
| *Truncatella angustata* | 100% |  |  |  |  | 1 |  |  |  |  |  |  |  |  |  | 1 | MN905872 |
| *Verticillium flavidum* | 99% |  | 1 |  |  |  |  |  |  |  |  |  |  |  |  | 1 | MN905873 |

| **Fungal Taxa** | **%**  **Identity** | **Level** | | | | | | | | | | | | | | **Total** | **Genbank #** |
| --- | --- | --- | --- | --- | --- | --- | --- | --- | --- | --- | --- | --- | --- | --- | --- | --- | --- |
|  |  | **7** | **8** | **9** | **10** | **11** | **12** | **15** | **17** | **18** | **21** | **22** | **23** | **25** | **27** |  |  |

| *Xenochalara juniperi* | 99% |  |  |  |  |  |  |  |  |  |  |  |  |  | 1 | 1 | MN905874 |
| --- | --- | --- | --- | --- | --- | --- | --- | --- | --- | --- | --- | --- | --- | --- | --- | --- | --- |
| ***Basidiomycota*** |  |  |  |  |  |  |  |  |  |  |  |  |  |  |  |  |  |
| *Postia floriformis* | 94% |  | 2 |  | 7 | 1 | 3 | 2 | 2 | 1 | 4 |  |  |  | 4 | 26 | MN905875 |
| *Sistotrema brinkmannii* | 100% |  | 3 | 2 | 1 |  | 5 |  |  |  | 4 | 1 |  | 10 |  | 26 | MN905876 |
| *Calocera cornea* | 87% |  | 9 |  | 4 | 1 | 1 |  |  | 1 | 4 |  |  | 1 | 3 | 24 | MN905877 |
| *Amyloathelia crassiuscula* | 84% |  |  |  |  |  | 3 | 1 | 1 |  |  |  |  | 1 | 5 | 11 | MN905878 |
| *Postia floriformis* | 99% |  |  |  |  | 1 | 6 |  |  |  |  |  |  |  | 2 | 9 | MN905879 |
| *Armillaria sinapina* | 99% | 4 |  |  | 2 |  | 1 |  |  |  |  |  |  |  |  | 7 | MN905880 |
| *Hyphodontia floccosa* | 99% |  |  | 2 | 1 |  |  |  | 1 |  |  |  |  |  | 1 | 5 | MN905881 |
| *Jaapia argillacea* | 98% |  |  |  |  |  | 1 |  |  |  | 4 |  |  |  |  | 5 | MN905882 |
| *Sistotrema oblongisporum* | 88% |  |  | 2 |  | 1 |  |  |  |  |  |  |  | 1 | 1 | 5 | MN905883 |
| *Coniophora puteana* | 100% |  |  |  |  |  | 2 |  | 2 |  |  |  |  |  |  | 4 | MN905884 |
| *Dacrymyces stillatus* | 99% |  |  |  |  |  | 1 |  |  | 1 |  |  |  |  | 2 | 4 | MN905885 |
| *Peniophorella pertenuis* | 99% |  |  |  |  |  |  |  |  | 1 | 3 |  |  |  |  | 4 | MN905886 |
| *Peniophorella pubera* | 99% |  |  |  |  |  | 2 |  |  |  | 1 |  |  |  | 1 | 4 | MN905887 |
| *Schizopora radula* | 99% |  |  |  |  |  |  |  |  |  |  |  |  | 1 | 3 | 4 | MN905888 |
| *Crustomyces subabruptus* | 99% |  |  |  |  |  |  |  |  |  |  |  |  |  | 3 | 3 | MN905889 |
| *Oligoporus balsameus* | 99% |  |  |  |  | 1 |  |  |  |  | 1 |  |  |  | 1 | 3 | MN905890 |
| *Physisporinus vitreus* | 96% |  | 3 |  |  |  |  |  |  |  |  |  |  |  |  | 3 | MN905891 |
| *Trametes palisotii* | 86% |  |  |  |  |  |  |  |  |  | 1 |  |  | 1 |  | 2 | MN905892 |
| *Athelia bombacina* | 99% |  |  |  |  |  |  |  |  |  | 1 |  |  |  |  | 1 | MN905893 |
| *Cerinosterus luteoalbus* | 96% |  |  |  |  |  |  |  |  |  | 1 |  |  |  |  | 1 | MN905894 |
| *Coprinellus micaceus* | 99% |  |  |  |  |  |  |  |  |  | 1 |  |  |  |  | 1 | MN905895 |
| *Peniophora cf. limitata* | 99% | 1 |  |  |  |  |  |  |  |  |  |  |  |  |  | 1 | MN905896 |
| *Physisporinus vitreus* | 99% |  |  |  | 1 |  |  |  |  |  |  |  |  |  |  | 1 | MN905897 |
| *Sebacina vermifera* | 85% |  |  |  |  |  |  |  | 1 |  |  |  |  |  |  | 1 | MN905898 |
| *Tapinella panuoides* | 99% |  |  |  |  |  |  |  |  |  | 1 |  |  |  |  | 1 | MN905899 |

| **Fungal Taxa** | **%**  **Identity** | **Level** | | | | | | | | | | | | | | **Total** | **Genbank #** |
| --- | --- | --- | --- | --- | --- | --- | --- | --- | --- | --- | --- | --- | --- | --- | --- | --- | --- |
|  |  | **7** | **8** | **9** | **10** | **11** | **12** | **15** | **17** | **18** | **21** | **22** | **23** | **25** | **27** |  |  |

| *Tyromyces fissilis* | 95% |  | 1 |  |  |  |  |  |  |  |  |  |  |  |  | 1 | MN905900 |
| --- | --- | --- | --- | --- | --- | --- | --- | --- | --- | --- | --- | --- | --- | --- | --- | --- | --- |
| ***Zygomycota*** |  |  |  |  |  |  |  |  |  |  |  |  |  |  |  |  |  |
| *Mortierella parvispora* | 98% |  |  | 1 | 2 | 3 | 3 | 1 | 3 |  | 5 |  | 2 | 2 | 5 | 27 | MN905901 |
| *Mortierella cf. gamsii* | 98% |  | 8 | 2 | 2 | 1 |  |  | 1 |  |  |  |  |  |  | 14 | MN905902 |
| *Mortierella hyalina* | 99% | 1 | 4 | 2 |  |  | 1 |  |  |  | 3 |  |  |  | 1 | 12 | MN905903 |
| *Mortierella basiparvispora* | 99% |  | 2 | 1 | 3 |  |  |  | 1 |  |  |  |  |  | 2 | 9 | MN905904 |
| *Mortierella hyalina* | 93% |  |  | 1 | 1 |  |  |  |  |  | 7 |  |  |  |  | 9 | MN905905 |
| *Mortierella gemmifera* | 99% |  | 1 | 1 | 1 |  |  |  |  |  | 2 |  |  |  |  | 5 | MN905906 |
| *Mortierella parvispora* | 96% |  |  |  |  |  |  |  |  |  | 4 |  |  |  | 1 | 5 | MN905907 |
| *Mortierella pulchella* | 99% |  | 1 |  | 2 |  |  |  |  |  | 1 |  |  | 1 |  | 5 | MN905908 |
| *Mortierella alpina* | 100% |  |  |  |  | 1 | 1 |  |  |  |  |  |  |  | 2 | 4 | MN905909 |
| *Mortierella minutissima* | 99% |  |  | 2 |  | 1 | 1 |  |  |  |  |  |  |  |  | 4 | MN905910 |
| *Umbelopsis isabellina* | 98% |  |  |  |  |  | 1 |  |  |  | 1 |  |  |  | 2 | 4 | MN905911 |
| *Mortierella verticillata* | 99% |  | 2 |  |  | 2 |  |  |  |  |  |  |  |  |  | 4 | MN905912 |
| *Mortierella beljakovae* | 91% | 2 |  | 1 |  |  |  |  |  |  |  |  |  |  |  | 3 | MN905913 |
| *Mortierella globulifera* | 85% |  |  |  | 1 | 1 | 1 |  |  |  |  |  |  |  |  | 3 | MN905914 |
| *Mortierella alpina* | 95% |  |  | 1 |  |  |  |  |  |  |  |  |  | 1 |  | 2 | MN905915 |
| *Mortierella amoeboidea* | 95% |  |  |  |  |  |  |  |  | 1 |  |  |  |  | 1 | 2 | MN905916 |
| *Mortierella dichotoma* | 100% |  |  |  |  |  |  |  |  |  |  |  |  |  | 2 | 2 | MN905917 |
| *Mortierella lignicola* | 91% |  |  |  |  |  |  |  |  |  | 2 |  |  |  |  | 2 | MN905918 |
| *Mortierella macrocystopsis* | 95% |  |  | 2 |  |  |  |  |  |  |  |  |  |  |  | 2 | MN905919 |
| *Mortierella macrocystopsis* | 100% |  |  | 1 |  |  |  |  |  |  | 1 |  |  |  |  | 2 | MN905920 |
| *Mortierella polycephala* | 99% |  |  |  |  |  |  |  |  |  |  |  |  |  | 2 | 2 | MN905921 |
| *Mortierella simplex* | 100% |  |  |  |  |  |  |  |  |  | 2 |  |  |  |  | 2 | MN905922 |
| *Helicostylum elegans* | 90% |  |  |  |  |  |  |  |  |  |  |  |  | 1 |  | 1 | MN905923 |
| *Helicostylum pulchrum* | 100% |  |  |  |  |  |  |  |  |  | 1 |  |  |  |  | 1 | MN905924 |
| *Mortierella angusta* | 100% |  |  |  |  |  |  |  |  |  | 1 |  |  |  |  | 1 | MN905925 |

| **Fungal Taxa** | **%**  **Identity** | **Level** | | | | | | | | | | | | | | **Total** | **Genbank #** |
| --- | --- | --- | --- | --- | --- | --- | --- | --- | --- | --- | --- | --- | --- | --- | --- | --- | --- |
|  |  | **7** | **8** | **9** | **10** | **11** | **12** | **15** | **17** | **18** | **21** | **22** | **23** | **25** | **27** |  |  |

| *Mortierella zychae* | 97% |  |  |  |  |  |  |  | 1 |  |  |  |  |  |  | 1 | MN905926 |
| --- | --- | --- | --- | --- | --- | --- | --- | --- | --- | --- | --- | --- | --- | --- | --- | --- | --- |
| *Muor flavus* | 88% |  |  |  |  |  |  |  |  |  |  |  |  |  | 1 | 1 | MN905927 |
| *Mucor heimalis* | 100% | 1 | 1 |  |  |  | 1 |  |  |  | 2 |  |  |  |  | 5 | MN905928 |
| *Mucor luteus* | 99% |  |  |  | 1 |  |  |  |  |  | 1 |  |  |  |  | 2 | MN905929 |
| *Mucor saturninus* | 99% |  | 1 |  |  |  |  |  |  |  |  |  |  |  | 1 | 2 | MN905930 |
| *Mucor zonatus* |  |  |  |  |  |  |  |  |  |  | 1 |  |  |  |  |  | MN905931 |
